# Associations between preoperative hypoalbuminemia and clinical outcomes following total hip or knee arthroplasty: a systematic review and meta-analysis

**DOI:** 10.1530/EOR-2025-0170

**Published:** 2026-04-07

**Authors:** Chen Yue, Yapeng Li, Lanbo Yang, Feng Li, Xiaolong Wu, Maoxiao Ma, Jiayi Guo

**Affiliations:** ^1^ Evidence-Based Medicine Center, Luoyang Orthopedic Hospital of Henan Province, Orthopedic Hospital of Henan Province, Luoyang, Henan Province, People’s Republic of China; ^2^ Henan Provincial Health Commission Key Laboratory of Bone Metabolism and Analysis, Luoyang Orthopedic Hospital of Henan Province, Orthopedic Hospital of Henan Province, Luoyang, Henan Province, People’s Republic of China; ^3^ The Third Clinical Medical College, Guangzhou University of Chinese Medicine, Guangzhou, Guangdong Province, China

**Keywords:** hypoalbuminemia, total hip or knee arthroplasty, clinical outcome

## Abstract

**Background:**

- Preoperative hypoalbuminemia is common in patients undergoing total hip or knee arthroplasty (THA/TKA). While it has been linked with poorer postoperative outcomes, there remains a paucity of systematic reviews or meta-analyses dedicated to conducting a comprehensive evaluation of this issue.

**Methods:**

- A comprehensive search was conducted across the PubMed, Embase, and Web of Science databases. Studies were selected and analyzed in adherence to the Meta-analysis of Observational Studies in Epidemiology (MOOSE) guidelines, and the quality of the evidence was evaluated using the Grading of Recommendations Assessment, Development, and Evaluation (GRADE) framework. Data were extracted and subjected to meta-analysis or qualitative synthesis for outcomes. This meta-analysis was registered in the PROSPERO database (NO. CRD42024581376).

**Results:**

- Fourteen studies involving 1,194,088 patients were included. Meta-analyses showed that preoperative hypoalbuminemia was associated with a greater risk of all-cause complications (OR: 2.89, 95% CI: 1.94–4.31), sepsis (OR: 2.54, 95% CI: 1.88–3.45), septic shock (OR: 3.44, 95% CI: 1.15–10.25), pneumonia (OR: 3.83, 95% CI: 3.14–4.67), urinary tract infection (OR: 1.59, 95% CI: 1.13–2.23), myocardial infarction (OR: 2.10, 95% CI: 1.47–2.98), superficial incisional infection (OR: 2.16, 95% CI: 1.56–3.00), periprosthetic joint infection (OR: 4.03, 95% CI: 2.15–7.53), wound dehiscence (OR: 1.68, 95% CI: 1.06–2.69), transfusion (OR: 1.84, 95% CI: 1.72–1.98), unplanned reoperation (OR: 1.60, 95% CI: 1.38–1.87), and mortality (OR: 7.14, 95% CI: 5.44–9.37).

**Conclusion:**

- Preoperative hypoalbuminemia is associated with an increased risk of numerous types of complications, transfusion, unplanned reoperation, and mortality after THA or TKA. Therefore, presurgical protocols formulated to deal with these unfavorable clinical outcomes may pay particular attention to this specific patient group.

## Introduction

Hypoalbuminemia, a marker of poor nutritional status, has emerged as a potential preoperative risk factor for more severe adverse outcomes across a range of surgical procedures (1, 2). Total hip or knee arthroplasty (THA/TKA) is a highly effective treatment for joints affected by advanced hip or knee diseases (3). Among patients scheduled for joint arthroplasty, preoperative hypoalbuminemia is prevalent (4). Several studies have shown a link between preoperative hypoalbuminemia and an elevated risk of postsurgical complications, such as sepsis (5), cardiovascular complications (6), pneumonia (7), venous thromboembolism events (8), and periprosthetic joint infections (5, 6, 9), following arthroplasty. Furthermore, preoperative hypoalbuminemia has been associated with an increased risk of unplanned readmission (10, 11) and even mortality (12).

Despite these findings, to our awareness, there is no published systematic review or meta-analysis that can carry out a detailed and critical analysis regarding how preoperative hypoalbuminemia influences the postsurgical outcomes for patients undergoing THA or TKA. Therefore, the aim of this study was to systematically review and meat-analyze the available literature to evaluate the impact of preoperative hypoalbuminemia on postoperative outcomes following total hip or knee arthroplasty. This study provides a THA/TKA-specific synthesis of the current evidence and evaluates multiple clinically relevant outcomes across different complication domains. We hypothesized that preoperative hypoalbuminemia is significantly associated with an increased incidence of postoperative complications and adverse outcomes compared to patients with normal preoperative albumin levels.

## Methods

This study was conducted in accordance with the ‘Meta-analysis of Observational Studies in Epidemiology’ (MOOSE) guidelines (13). The present study was registered in the PROSPERO database with the registration number CRD42024581376*.* Ethical approval for this study was considered unnecessary because it involved a review of peer-reviewed literature and did not directly involve patient-level data.

PICO definition: population (P): adult patients undergoing primary total hip arthroplasty (THA) or total knee arthroplasty (TKA); intervention/exposure (I): preoperative hypoalbuminemia, as defined by the original studies; comparator (C): patients with normal preoperative serum albumin levels; and outcomes (O): postoperative complications and other adverse clinical outcomes following THA or TKA.

### Search strategy

Two independent investigators systematically searched PubMed, Embase, and Web of Science using combinations of MESH terms and keywords related to hypoalbuminemia and total hip/knee arthroplasty. The detailed search strategies for each of these databases can be found in Supplemental Fig. 1 (see section on Supplementary materials given at the end of the article). In addition, references lists of relevant articles were manually searched to identify additional eligible studies. No language restrictions were applied, and non-English studies were translated with the assistance of language experts when necessary.

### Study selection

Studies were considered to be suitable for inclusion in this meta-analysis if they were cohort or case–control studies investigating the impact of preoperative hypoalbuminemia compared with normal serum albumin levels on postoperative outcomes following THA or TKA. Studies were excluded if they were not controlled (e.g. they were case or case-series reports), if the full text of the article was unavailable, if they involved patients undergoing revision hip or knee arthroplasty or hemi-hip or unicompartmental knee arthroplasty, or if they evaluated the impact of postoperative hypoalbuminemia on outcomes after primary arthroplasty.

Potential studies were imported into Endnote X9 software (Thomson Scientific, USA), and duplicate records were removed. Two authors independently excluded irrelevant studies based on a review of titles and abstracts. The same two authors then read the full texts of the remaining articles to produce a final list of studies. Any discrepancies between the two authors were resolved by discussion with a third author.

### Data extraction and assessment of quality of study and evidence

Two independent investigators extracted a range of data from the included studies, encompassing the first author’s name, publication year, country of origin, surgery type, sample size, study design, and follow-up duration (any postoperative time point). The outcomes of interest were prespecified and categorized into primary and secondary outcomes as follows: i) primary outcome: all-cause complications; ii) secondary outcomes: systemic complications, including sepsis, septic shock, pneumonia, stroke, cardiac arrest, myocardial infarction, urinary tract infections, acute renal failure, and venous thromboembolism events, and surgical complications, such as superficial incisional infections, periprosthetic joint infections, wound dehiscence, periprosthetic fractures, and dislocations; and iii) additional outcomes, such as transfusion requirements, mortality, unplanned reoperation, and unplanned readmission. The investigators would contact the authors of the included studies via email to request data of interest that were not fully reported in the original articles.

To assess the methodological quality of each study, we utilized the validated and widely adopted Newcastle–Ottawa scale (NOS) (14). This scoring system evaluates observational studies based on three criteria: cohort study groups (ranging from 0 to 4 points), comparability of cases and controls (0–2 points), and the ascertainment method for the outcome of interest (0–3 points). With a maximum total of nine points, only studies deemed to be of moderate or high methodological quality (scoring at least five points) were included in this meta-analysis. Furthermore, we assessed the quality of evidence using the Grading of Recommendations Assessment, Development, and Evaluation (GRADE) framework within GRADEpro 3.6 software, provided by the GRADE Working Group (15). The certainty of evidence was independently evaluated by two reviewers. The quality grade was categorized into four levels: very low, low, medium, and high. Two researchers independently evaluated the quality of evidence, and any disagreements were resolved through discussion with a third researcher. The certainty of evidence was assessed using the Grading of Recommendations Assessment, Development, and Evaluation (GRADE) approach. Observational studies were initially rated as low certainty. The certainty of evidence was downgraded for inconsistency when statistical heterogeneity was substantial (*I^2^* = 50–75%, downgraded by one level) or very high (*I^2^* > 75%, downgraded by two levels). The certainty was downgraded for indirectness when pooled analyses combined patients undergoing total hip arthroplasty and total knee arthroplasty, as such analyses may limit procedure-specific applicability. Imprecision was assessed based on the width of confidence intervals, and the certainty was downgraded when confidence intervals crossed the line of no effect. Upgrading of the certainty of evidence was applied in observational studies when a large magnitude of effect was observed, with one-level upgrading for large effects (OR > 2) and two-level upgrading for very large effects (OR > 5).

### Statistical analysis

If data could not be directly compared across studies or if the studies reported insufficient data, the outcomes were synthesized qualitatively. For studies that reported sufficient and comparable data, a meta-analysis was conducted using a variance-based random-effects model (the DerSimonian–Laird method) and displayed as forest plots using STATA 18 software (Stata Corp, USA). All variables of interest in this study were dichotomous, and the pooled risk estimates were expressed as odds ratio (OR) with 95% confidence interval (CI). Results were considered statistically significant if they were associated with a two-tailed *P*-value of less than 0.05.

The heterogeneity of meta-analyzed outcomes was assessed using the *I^2^* test and was considered absent if *I^2^* was no larger than 25.0%, low if *I^2^* = 25.1–50.0%, moderate if *I^2^* = 50.1–75.0%, or high if *I^2^* = 75.1–100.0%. A subgroup analysis was performed based on the study type (cohort versus case–control) to assess the impact of the research design on the robustness of the results. We performed a series of sensitivity analyses. When heterogeneity was present, a ‘leave-one-out’ sensitivity analysis was carried out to explore its source. In addition, the Hartung–Knapp method was conducted to obtain conservative confidence interval, which was suggested to address potential issues with type I error rate from small numbers of studies.

## Results

### Search results and characteristics of the included studies

**Figure 1:**
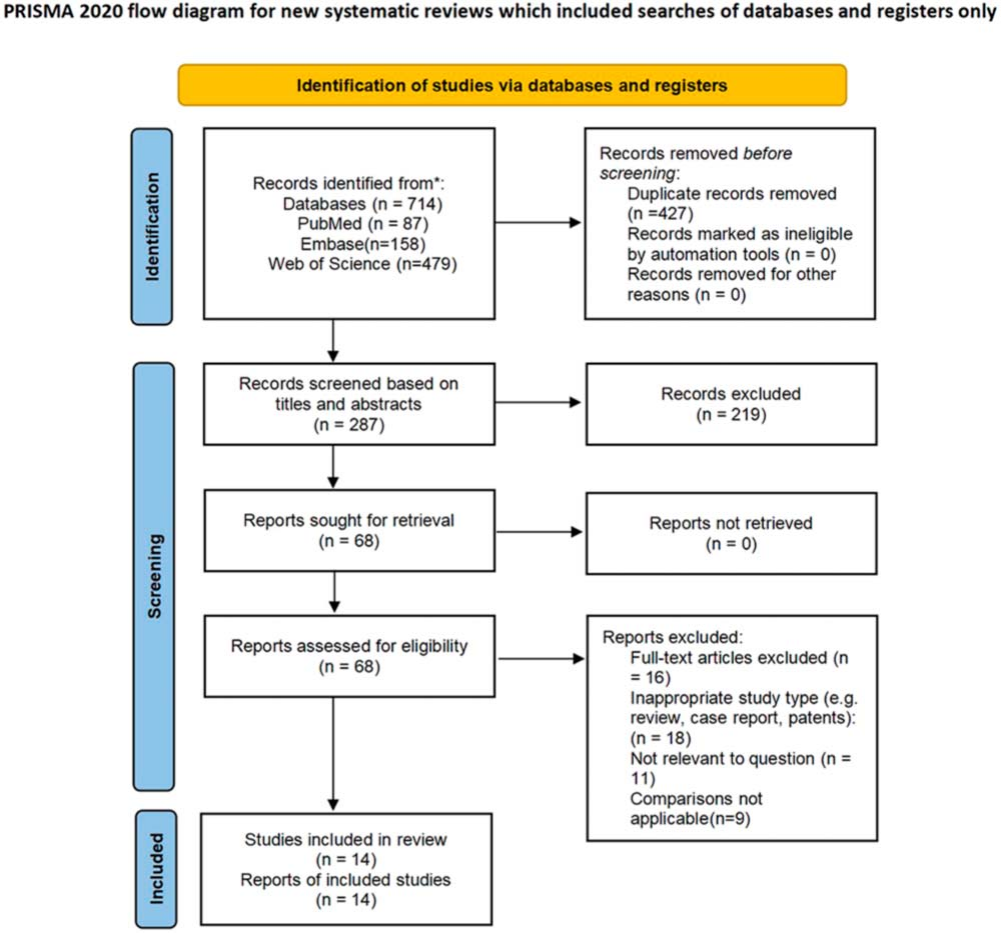
Flow chart of study identification, screening, and selection.

A total of 714 articles were retrieved in the initial search, and no additional records were found by manually searching references. After removal of 427 duplicate records and exclusion of another 219 records as irrelevant based on review of titles and abstracts, the full texts of the remaining 68 articles were read. Based on the inclusion and exclusion criteria, the remaining 14 studies involving 1,194,088 patients were included in the systematic review (Fig. 1).

The review included six cohort studies (11, 16, 17, 18, 19, 20) and eight case–control studies (5, 8, 9, 10, 21, 22, 23, 24). All studies involved patient populations at a single medical center and were published within the past 10 years, including ten studies published within the past five years (5, 9, 10, 11, 16, 17, 18, 19, 21). Scores of the included studies on the Newcastle–Ottawa scale ranged from six to eight points. Preoperative hypoalbuminemia in all studies was defined as serum albumin <3.5 g/dL.

The detailed description of study characteristics, participants, and summary of outcomes is shown in Table 1 and Supplemental Table 1.

**Table 1:** Description of study characteristics and participants.

Study	Type of arthroplasty	Country	Number of patients			Follow-up	Study design	NOS score
			Total	HA	NA			
Elliott *et al.* (10)	Hip or knee	USA	79,784	3,959	75,825	Unclear	Case–control	7
Lung *et al.* (17)	Hip	USA	275,107	7,728	267,379	Unclear	Cohort	7
Lung *et al.* (18)	Hip	USA	275,107	7,728	267,379	Unclear	Cohort	7
Abella *et al.* (11)	Hip or knee	USA	31,851	718	31,133	30 days	Cohort	8
Johnson *et al.* (19)	Knee	USA	45,152	2,555	42,597	90 days	Cohort	7
Newman *et al.* (5)	Hip	USA	1,667	569	1,098	30 days	Case–control	7
Guo *et al.* (16)	Hip or knee	China	288	110	178	12 months	Cohort	8
Sloan *et al.* (21)	Knee	USA	108,601	4,327	104,274	30 days	Case–control	7
Man *et al.* (9)	Knee	China	624	17	607	Unclear	Case–control	7
Fryhofer *et al.* (22)	Hip or knee	USA	173,579	7,391	166,188	30 days	Case–control	6
Ryan *et al.* (8)	Hip or knee	USA	128,412	564	122,948	30 days	Case–control	7
Haro-Gómez *et al.* (23)	Hip	Mexico	75	31	44	Unclear	Case–control	6
Bohl *et al.* (20)	Hip or knee	USA	49,603	1,964	47,639	30 days	Cohort	8
Walls *et al.* (24)	Hip	USA	24,238	1,122	23,116	30 days	Case–control	6

NOS, Newcastle–Ottawa scale; HA, hypoalbuminemia; and NA, normal albumin.

### All-cause complications

**Figure 2:**
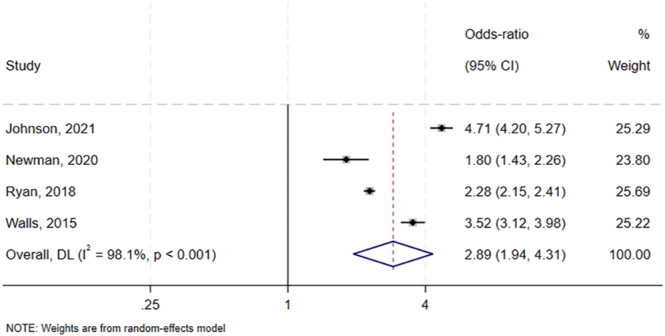
Forest plot of the potential association between preoperative hypoalbuminemia and risk of all-cause complications after primary total hip or knee arthroplasty. CI, confidence interval.

Meta-analyses of four trials involving 196,883 patients (5, 8, 19, 24) linked preoperative hypoalbuminemia with a higher risk of all-cause complications (OR: 2.89, 95% CI: 1.94–4.31). This result was associated with high heterogeneity (*I^2^* = 98.1%, *P* < 0.001; Fig. 2), and the quality of evidence (GRADE) was very low.

### Systemic complications

**Figure 3:**
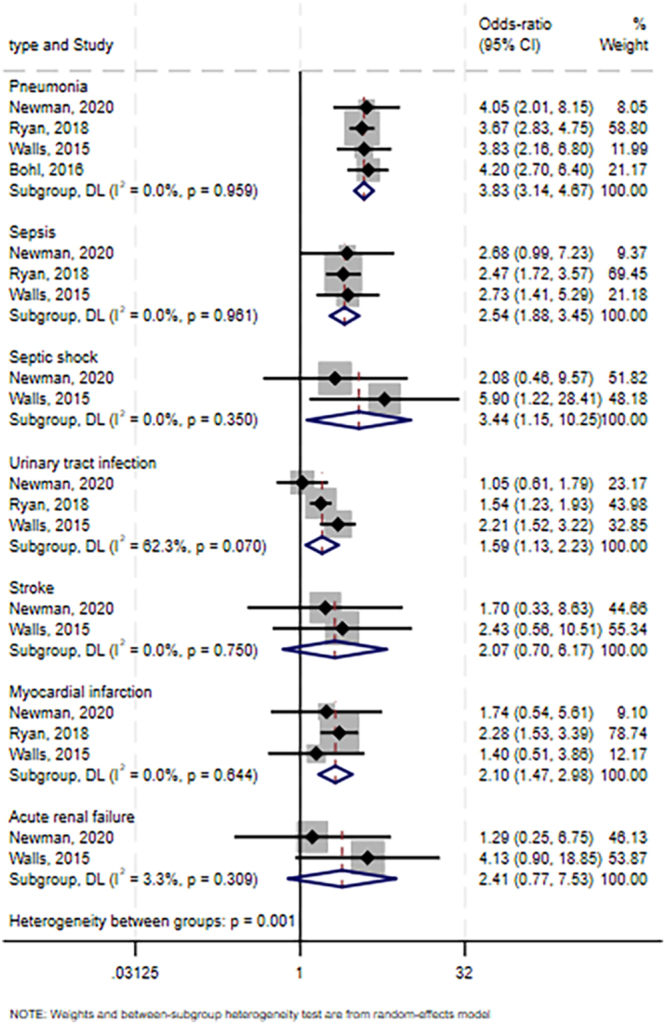
Forest plot of the potential association between preoperative hypoalbuminemia and risk of systemic complications after primary total hip or knee arthroplasty. CI, confidence interval.

Meta-analyses linked preoperative hypoalbuminemia with the following specific systemic complications (Fig. 3): sepsis (OR: 2.54, 95% CI: 1.88–3.45; *I^2^* = 0%; GRADE: low; three trials involving 154,317 patients (5, 8, 24)), septic shock (OR: 3.44, 95% CI: 1.15–10.25; *I^2^* = 0%; GRADE: low; two trials involving 25,905 patients (5, 24)), pneumonia (OR: 3.83, 95% CI: 3.14–4.67; *I^2^* = 0%; GRADE: low; four trials involving 203,920 patients (5, 8, 20, 24)), myocardial infarction (OR: 2.10, 95% CI: 1.47–2.98; *I^2^* = 0%; GRADE: low; three studies involving 154,317 patients (5, 8, 24)), and urinary tract infection (OR: 1.59, 95% CI: 1.13–2.23; *I^2^* = 62.3%; GRADE: very low; three studies involving 154,317 patients (5, 8, 24)). In contrast, meta-analyses did not detect a significant association between preoperative hypoalbuminemia and risk of stroke (OR: 2.07, 95% CI: 0.70–6.17; *I^2^* = 0%; GRADE: very low; two studies involving 25,905 patients (5, 24)) or risk of acute renal failure (OR: 2.41, 95% CI: 0.77–7.53; *I^2^* = 3.3%; GRADE: very low; two studies involving 25,905 patients (5, 24)).

We were unable to perform a meta-analysis on data from studies regarding the outcomes of venous thromboembolism events, owing to considerable heterogeneity in the types of thrombosis and the assessment time points. One study involving 128,412 patients linked preoperative hypoalbuminemia to a significantly higher risk of venous thromboembolic events (8), while this association did not achieve significance in another study involving 75 patients (23). Two studies involving 25,905 patients linked preoperative hypoalbuminemia to a significantly greater risk of pulmonary embolism but not deep vein thrombosis (5, 24).

Only one of the included studies, which involved 1,667 patients, reported risk of cardiac arrest, which was significantly higher among those with preoperative hypoalbuminemia than among those without it (1.8 versus 0.4%) (5).

### Surgical complications

**Figure 4:**
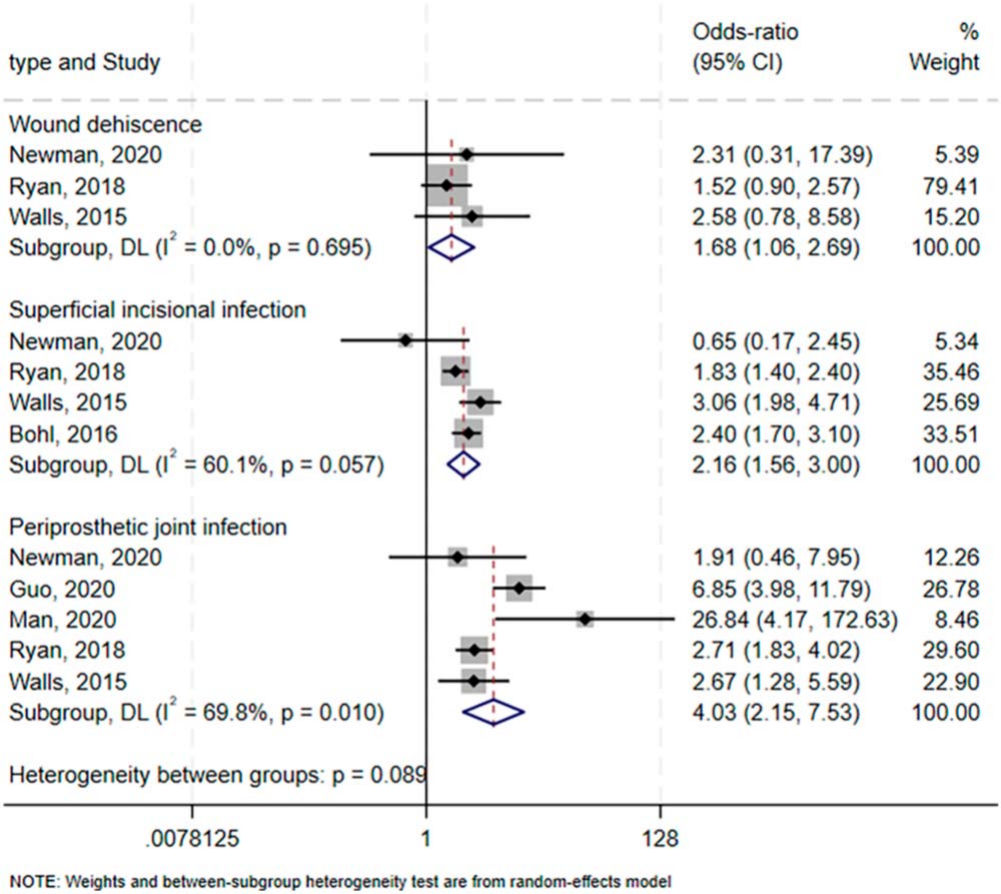
Forest plot of the potential association between preoperative hypoalbuminemia and risk of surgical complications after primary total hip or knee arthroplasty. CI, confidence interval.

Meta-analyses linked preoperative hypoalbuminemia to the following surgical complications (Fig. 4): wound dehiscence (OR: 1.68, 95% CI: 1.06–2.69, *I^2^* = 0%; GRADE: very low; three studies involving 154,317 patients (5, 8, 24)), superficial incisional infection (OR: 2.16, 95% CI: 1.56–3.00; *I^2^* = 60.1%; GRADE: very low; four trials involving 203,920 patients (5, 8, 20, 24)), and periprosthetic joint infection (OR: 4.03, 95% CI: 2.15–7.53 *I^2^* = 69.8%; GRADE: very low; five studies involving 155,229 patients (5, 8, 9, 16, 24)).

Conducting a meta-analysis on the outcomes of periprosthetic fracture and dislocation was not feasible due to the insufficient availability of data. Two studies involving 275,107 individuals linked preoperative hypoalbuminemia to a significantly greater risk of periprosthetic fracture (18) or dislocation (17).

### Transfusion, mortality, unplanned reoperation, and unplanned readmission

**Figure 5:**
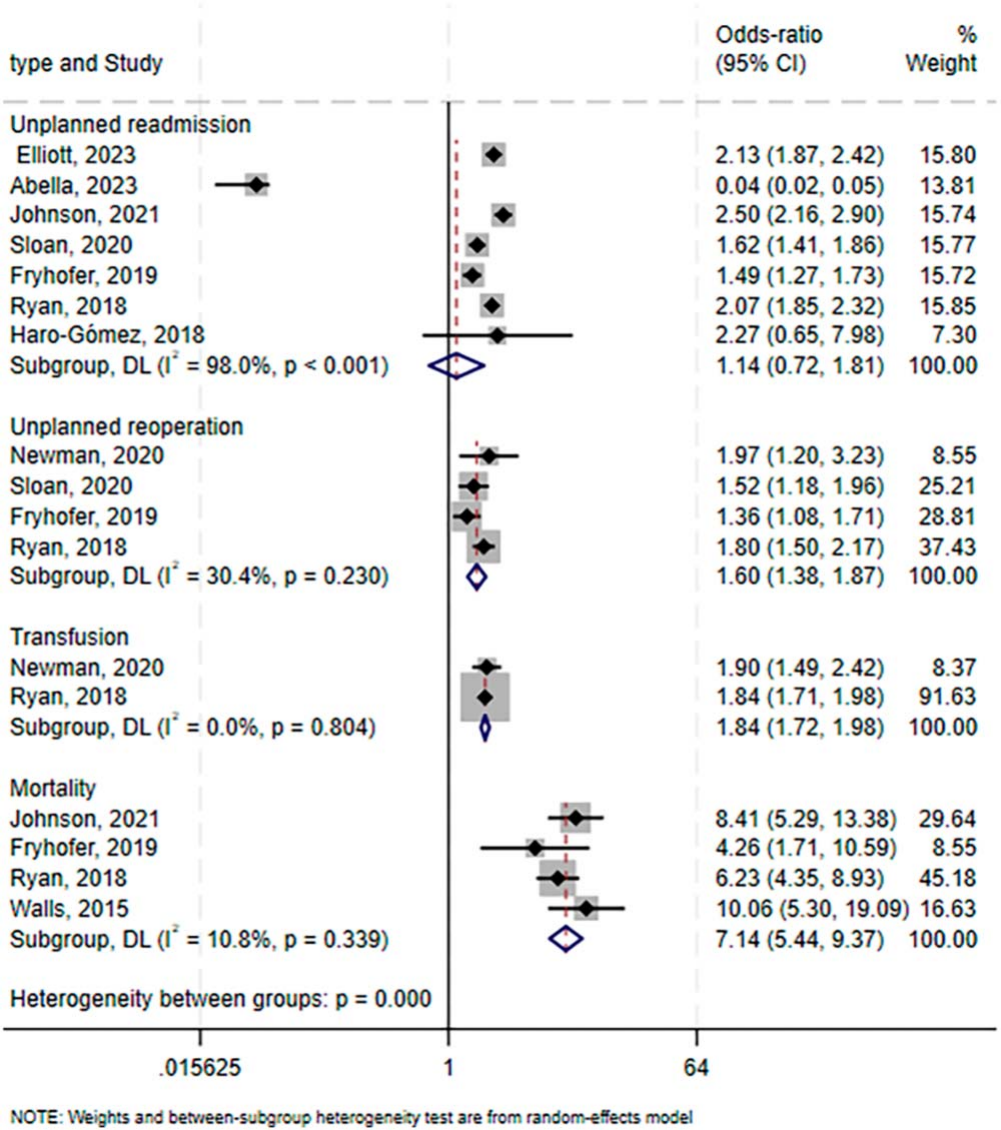
Forest plot of the potential association between preoperative hypoalbuminemia and risk of additional outcomes after primary total hip or knee arthroplasty. CI, confidence interval.

Meta-analyses also showed that preoperative hypoalbuminemia is associated with an increased risk of transfusion (OR: 1.84, 95% CI: 1.72–1.98; *I*^2^ = 0%; GRADE: very low; two studies involving 130,079 patients (5, 8)), mortality (OR: 7.14, 95% CI: 5.44–9.37, *I^2^* = 10.8%; GRADE: moderate; four studies involving 371,381 patients (8, 19, 22, 24)), and unplanned reoperation (OR: 1.60, 95% CI: 1.38–1.87; *I^2^* = 30.4%; GRADE: very low; four studies involving 398,309 patients (5, 8, 21, 22)). In contrast, meta-analyses did not detect a significant association between preoperative hypoalbuminemia and risk of unplanned readmission (OR: 1.14, 95% CI: 0.72–1.81; *I^2^* = 98%; GRADE: very low; seven studies involving 547,760 patients (8, 10, 11, 19, 21, 22, 23)) (Fig. 5).

The GRADE classifications and relevant descriptions for all the aforementioned outcomes are presented in Supplemental Table 2.

### Subgroup analysis

The outcomes of subgroup analyses, stratified by study type (cohort studies versus case–control studies), are consistent in indicating that preoperative hypoalbuminemia elevates the risk of all-cause complications, pneumonia, superficial incisional infections, periprosthetic joint infections, and mortality (Supplemental Fig. 2).

### Sensitivity analysis

Repeating meta-analyses after leaving out individual studies led to similar results as including all studies for the outcomes of pneumonia, superficial incisional infection, periprosthetic joint infection, urinary tract infection, unplanned readmission, and mortality (Supplemental Table 3). Sensitivity analysis failed to identify individual studies contributing to the high heterogeneity in the meta-analysis of all-cause complications, which may reduce its reliability. Furthermore, regarding outcomes such as all-cause complications, myocardial infarction, pneumonia, sepsis, periprosthetic joint infection, superficial incisional infection, mortality, unplanned reoperation, and unplanned reoperation readmission, the results of meta-analyses remained consistent even after adjusting confidence intervals using the Hartung–Knapp method. These findings aligned with those obtained via the Wald-type method (Supplemental Table 4).

## Discussion

Our systematic review and meta-analysis indicates that the available evidence supports a link between poor preoperative nutritional status, as reflected in hypoalbuminemia, and a greater risk of all-cause complications, several kinds of systemic or surgical complications, transfusion, reoperation, readmission, and even mortality after primary total hip or knee arthroplasty. Our findings support consensus guidance about the dangers of malnourishment in patients undergoing total hip or knee arthroplasty (25).

Hypoalbuminemia decreases the oncotic pressure of the blood, resulting in edema in the surrounding tissues. This might delay local wound healing process by impeding the effective accumulation of albumin, fibrinogen, immunoglobulins, electrolytes, and nutrients into wounds and newly growing tissues (26, 27, 28). Albumin plays a crucial role in facilitating neutrophil degranulation and phagocytosis through its interactions with various inflammatory mediators (29, 30), and it also promotes antibody production. Consequently, its deficiency may compromise the efficacy of the systemic immune system (28, 31, 32). These mechanisms offer potential explanations for why preoperative hypoalbuminemia elevates the risk of not only local infections, such as superficial incisional infections and periprosthetic joint infections, but also systemic infections, including pneumonia, sepsis, septic shock, and urinary tract infections, after THA or TKA. Our analysis justifies further research into whether and how poor nutritional status contributes directly to the infection-related complications whose risk appears to increase.

Hypoalbuminemia may contribute to stroke, cardiac arrest, myocardial infarction (33, 34, 35), and other cardio-cerebrovascular complications because albumin is the most abundant anti-oxidant in whole blood. Its deficiency can lead to oxidative stress, inflammatory responses, and platelet aggregation (36, 37, 38, 39, 40), all of which can exacerbate cardiovascular conditions. By contributing to myocardial edema, hypoalbuminemia may also exacerbate some cardiovascular conditions, particularly ischemic heart disease, and increase risk of heart failure (36). Our meta-analysis has identified an association between preoperative hypoalbuminemia and a higher risk of myocardial infarction in patients undergoing arthroplasty. However, due to a lack of sufficient data, we are unable to determine its association with other cardiovascular complications, such as cardiac arrest and stroke. This represents an area for future research.

We were unable to meta-analyze risk of venous thromboembolic events because the included studies varied in how they defined such events and the time points when they measured them. Such events, which include deep vein thrombosis and pulmonary embolism, are a frequent complication after orthopedic surgery (37, 38), and albumin in the blood may help prevent such events through their ability to reduce oxidative stress and inhibit inflammatory responses and platelet aggregation (39). Deficiency of albumin may lead to inappropriate aggregation of platelets through a mechanism related to Nox2 (40). Future studies should focus on clarifying whether and how preoperative hypoalbuminemia contributes to risk of venous thromboembolic events after arthroplasty. Such studies should standardize thromboprophylaxis and how and when events are diagnosed in order to ensure that outcome data can be pooled.

Hypoalbuminemia, as an indicator of poor nutritional status, is associated with a reduced mass and strength of muscles (41) and bone (42, 43). The resulting difficulties in movement may increase the risk of dislocation or fracture (44, 45). In this review, our inclusion of limited evidence indicates a potential link between preoperative hypoalbuminemia and a greater risk of postoperative dislocation and periprosthetic fractures. However, the insufficient data precluded the conduct of a meta-analysis, thereby undermining the robustness of our findings. Future studies should explore whether and how preoperative hypoalbuminemia contributes to such risk, again using standardized protocols to ensure data poolability. This question is particularly important to examine because postoperative dislocation and periprosthetic fractures are not only independent postoperative complications, but also associated with higher rates of thromboembolic events, infections, bleeding, rehospitalization, reoperations, and even mortality. The elevated occurrence of numerous systemic and surgical site complications linked to preoperative hypoalbuminemia may help explain why it is associated with a higher risk of all-cause complications and reoperations. Notably, the results of our meta-analysis disclose that patients with preoperative hypoalbuminemia are at a significantly greater risk of postoperative mortality compared to those without preoperative hypoalbuminemia. These findings highlight the crucial necessity of conducting a comprehensive preoperative assessment and addressing hypoalbuminemia prior to surgery.

Our study also has several limitations. First, the findings of our study regarding the outcome of all-cause complications should be interpreted with caution due to the high heterogeneity among studies, for which the pooled risk estimate may be less reliable. Second, several outcomes could not be meta-analyzed due to significant heterogeneity across studies or inadequate data availability. As a result, our reliance solely on qualitative synthesis for these outcomes necessitates acknowledging a lesser degree of robustness in our conclusions. Third, up to now, there is still a lack of evidence to support whether preoperative hypoalbuminemia in THA/TKA patients would lead to certain potential adverse outcomes, including complications such as edema, pleural effusions, and intestinal obstruction, and self-reported outcomes, such as pain and function. Thus, we were unable to identify any valuable data regarding these outcomes to undertake a meta-analysis or systematic review, which constitutes a limitation in our study. In the future, large-scale cohort studies should place greater emphasis on reporting these outcomes so as to provide clinical evidence for medical staff. Fourth, as nearly all the included original studies reported only unadjusted effect sizes, we could only use such unadjusted data for meta-analyses. This may cause biases in the results due to confounding factors, such as age and comorbidities, which is another limitation of this review. Future studies should report both adjusted and unadjusted effect sizes to improve the control of confounding factors in subsequent meta-analyses. Then, separate subgroup analyses for total hip arthroplasty and total knee arthroplasty could not be performed, as several included studies reported outcomes for THA and TKA as a combined cohort without providing procedure-specific data. This limitation may contribute to indirectness in the certainty of evidence and should be addressed in future studies with more granular reporting. Finally, due to the lack of available data, we were unable to assess whether preoperative hypoalbuminemia exhibits a dose–response association with these adverse outcomes. However, this systematic review and meta-analysis provides a THA/TKA-specific synthesis of the available evidence, integrating multiple clinically relevant postoperative outcomes, and represents one of the most comprehensive evaluations to date on the impact of preoperative hypoalbuminemia in this patient population. Nevertheless, we conducted subgroup analyses and sensitivity analyses and employed the GRADE system to evaluate the quality of evidence. Consequently, our findings can render effective assistance to medical staff, enabling them to gain a more thorough understanding of this clinical issue.

In addition to poor nutritional status, hypoalbuminemia may arise from other factors, such as systemic inflammation, liver or kidney dysfunction, and acute illness (26, 46, 47, 48). These alternative causes were not consistently excluded in the included studies. Nevertheless, in most cases, preoperative hypoalbuminemia likely reflected a consequence of poor nutrition. This limitation should be considered when interpreting the associations observed in our review, as other underlying conditions may have contributed to the outcomes.

In conclusion, our assessment of more than 1.1 million patients supports the associations between preoperative hypoalbuminemia with a greater risk of numerous types of complications, transfusion, unplanned reoperation, and even mortality in patients undergoing THA or TKA. Surgeons should be aware of these elevated risks. Meanwhile, presurgical protocols formulated to deal with these unfavorable clinical outcomes may pay particular attention to this specific patient group. Future research should concentrate on the question of whether correcting preoperative hypoalbuminemia can decrease the probability of these adverse postoperative outcomes.

## Supplementary materials



## ICMJE Statement of Interest

All authors declare no personal financial interests, institutional/commercial relationships, academic competition conflicts, or other potential conflicts related to this research.

## Funding Statement

This work was funded by the National Project on Traditional Chinese Medicine (GZK-KJS-2023-012) and National Project of Traditional Chinese Medicine’s Evidence-Based Capacity Building (2019XZZX-GK004).

## Author contribution statement

CY conceived the study and wrote the original draft of the manuscript. YLi performed formal analysis, acquired software, and wrote the original draft of the manuscript. LY designed the methodology. CW and FL curated the data and acquired software. YLiu designed the methodology and administered the project. XW supervised the study and wrote, reviewed, and edited the manuscript. MM conceived the study and wrote, reviewed, and edited the manuscript. JG conceived the study, acquired funding, administered the project, supervised the study, and wrote, reviewed, and edited the manuscript.

## Data statement

The data are available from the corresponding author on reasonable request.
