# Supplementary material for: Associations between preoperative hypoalbuminemia and clinical outcomes following total hip or knee arthroplasty: a systematic review and meta-analysis
Source: EFORT Open Rev. 2026 Apr 7;11(4):268–76. doi: 10.1530/EOR-2025-0170 (PMC13087879; doi:10.1530/EOR-2025-0170)
Supplement: Supplementary file 1 [file supplementary_materials.pdf]

Supplemental Figure 1. A systematic search was conducted across PubMed(A), Embase(B), and Web of Science(C).

Supplemental Figure 1A.

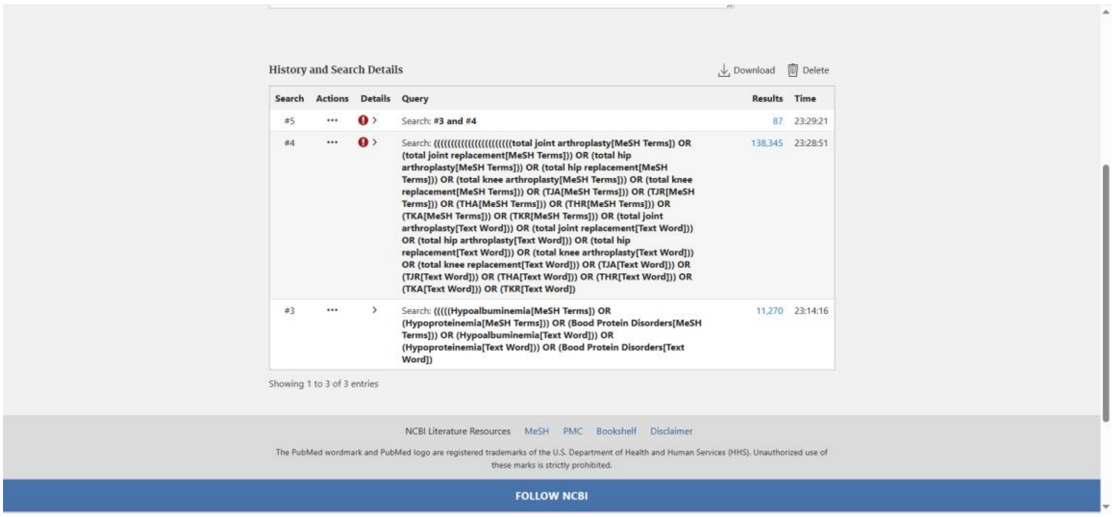

Supplemental Figure 1B.

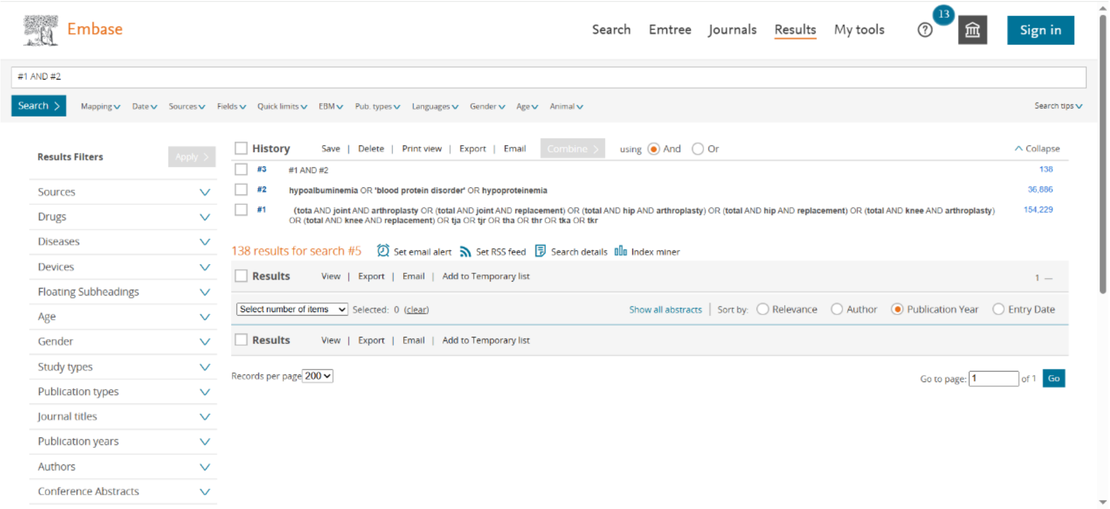

Supplemental Figure 1C.

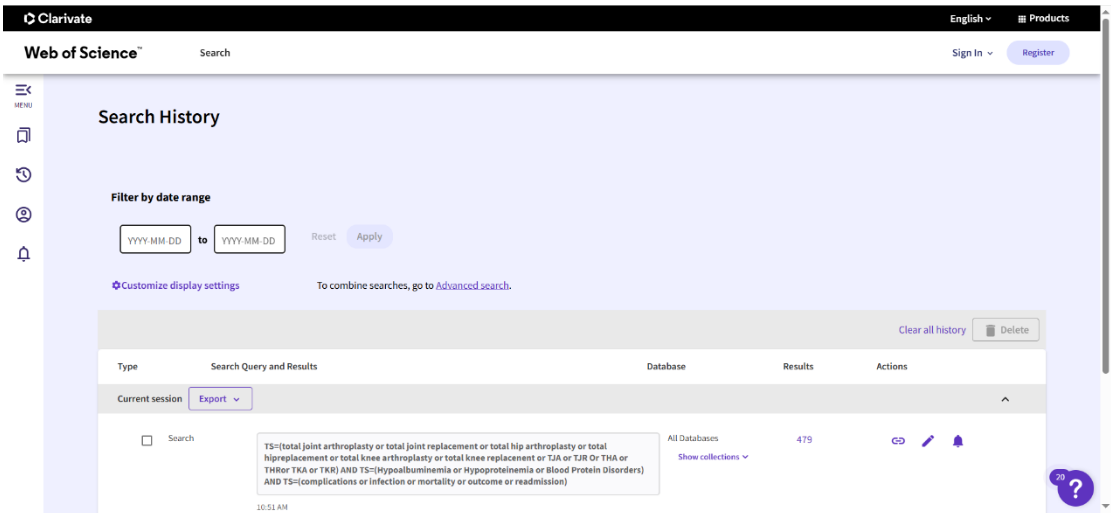

Supplemental Figure 2. Forest plot of subgroup analysis for all-cause complications (A), pneumonia (B), superficial incisional infection (C), periprosthetic joint infection (D), unplanned readmission (E), and mortality (F) based on study type.

Supplemental Figure 2A.

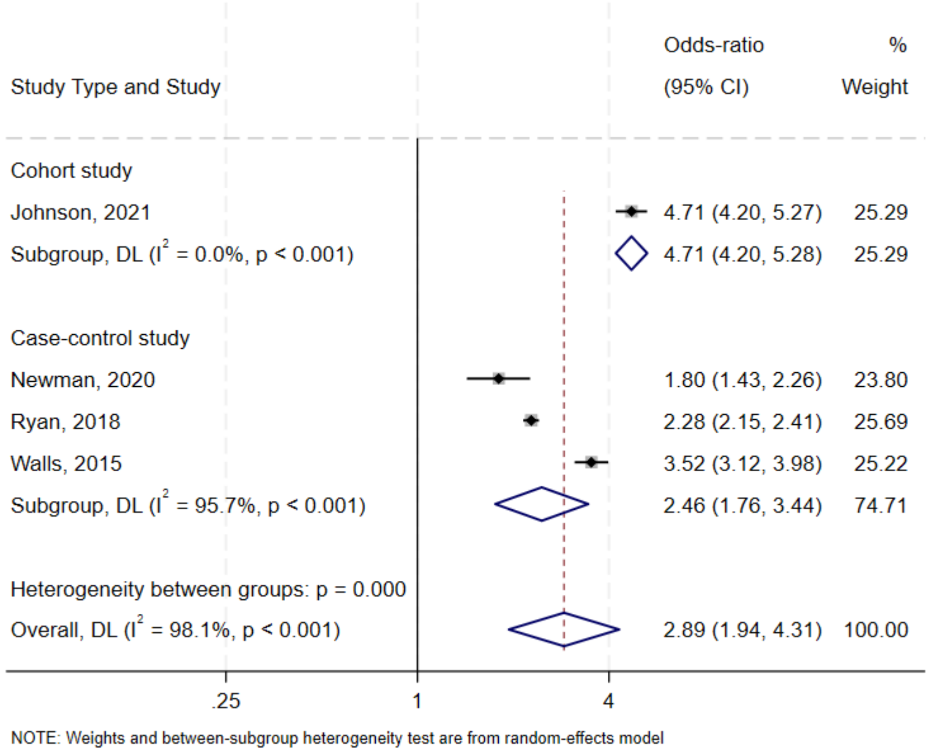

Supplemental Figure 2B.

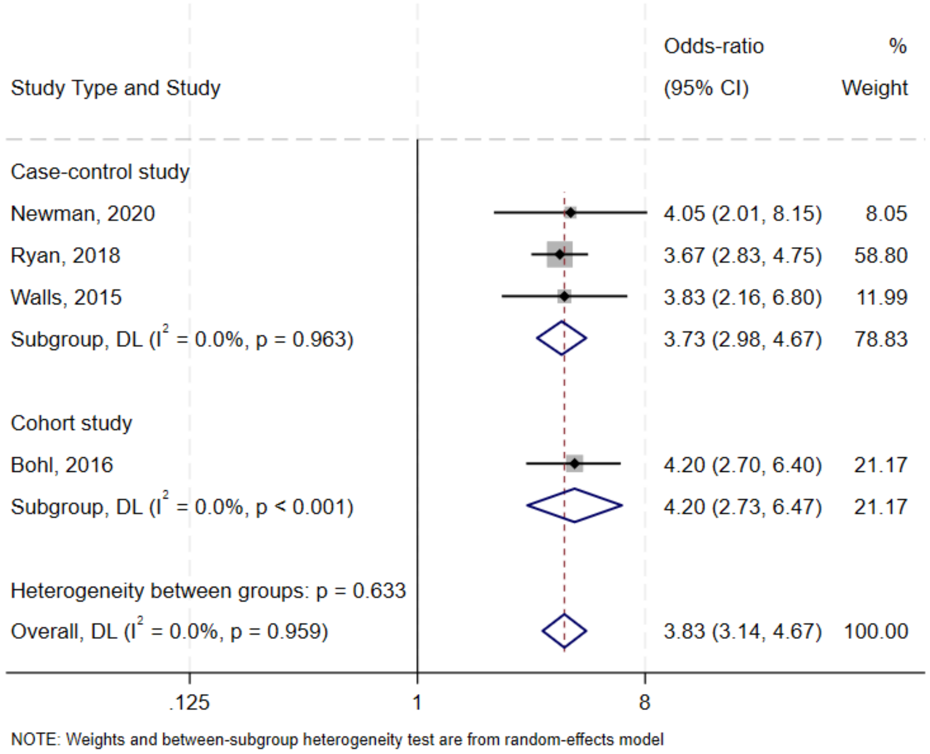

Supplemental Figure 2C.

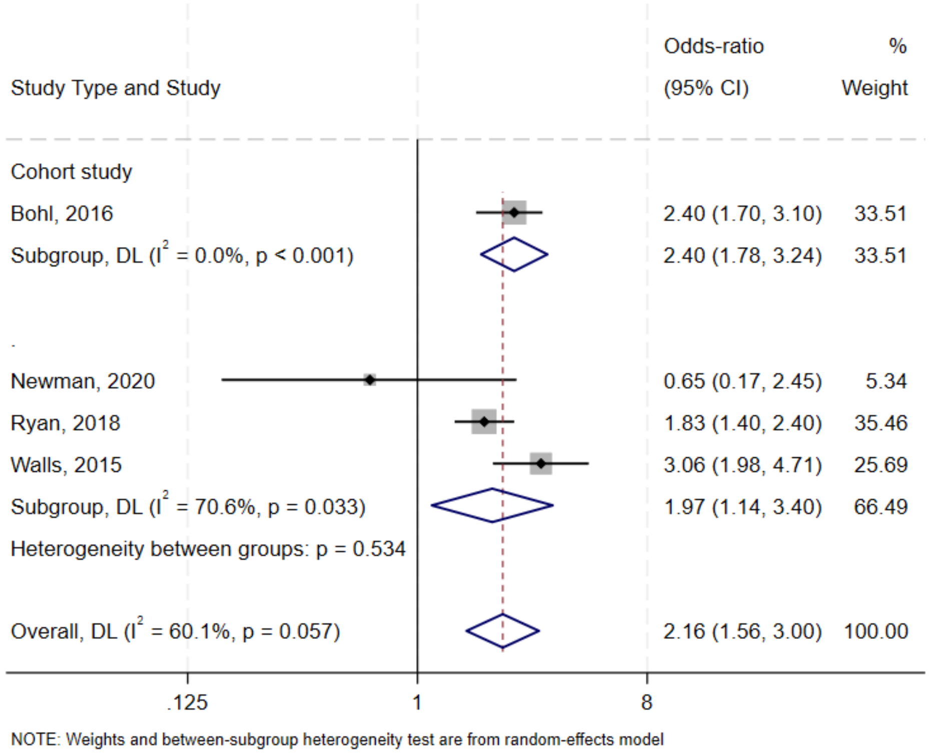

Supplemental Figure 2D.

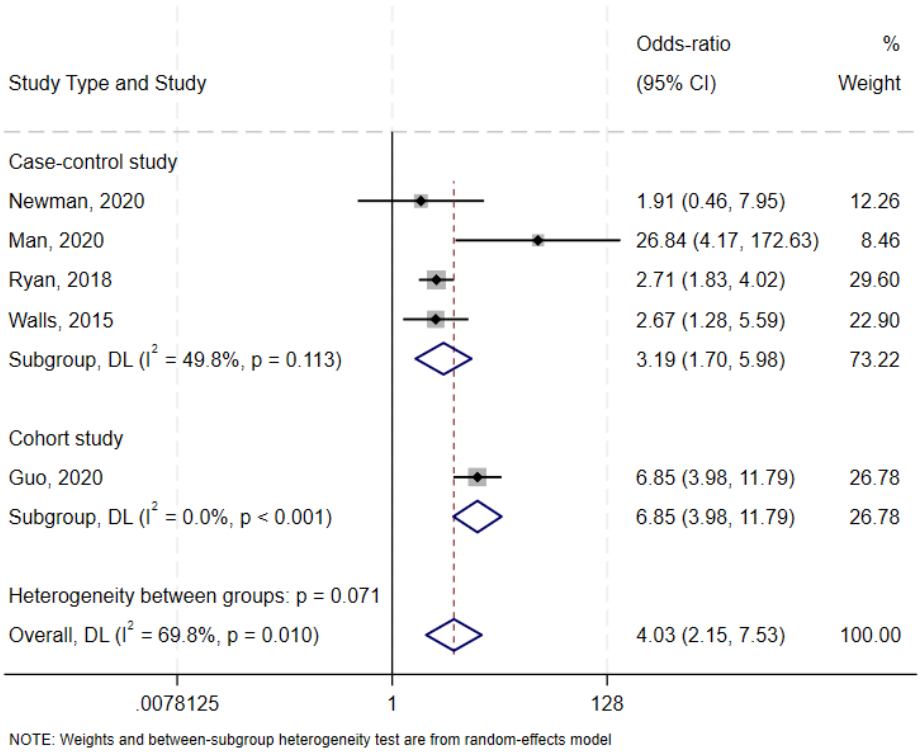

Supplemental Figure 2E.

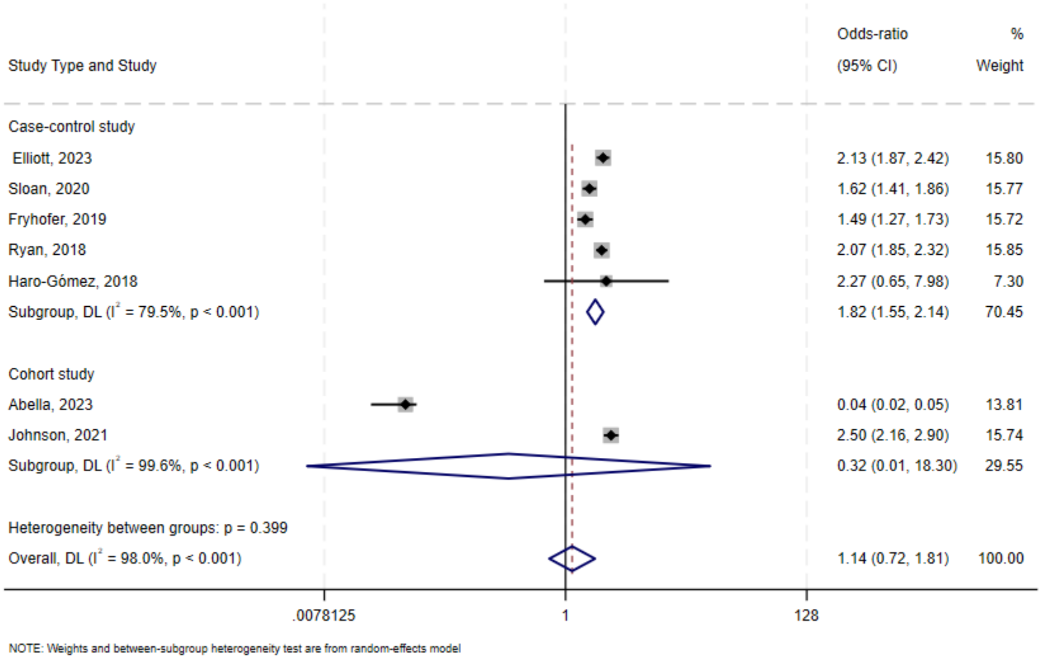

Supplemental Figure 2F.

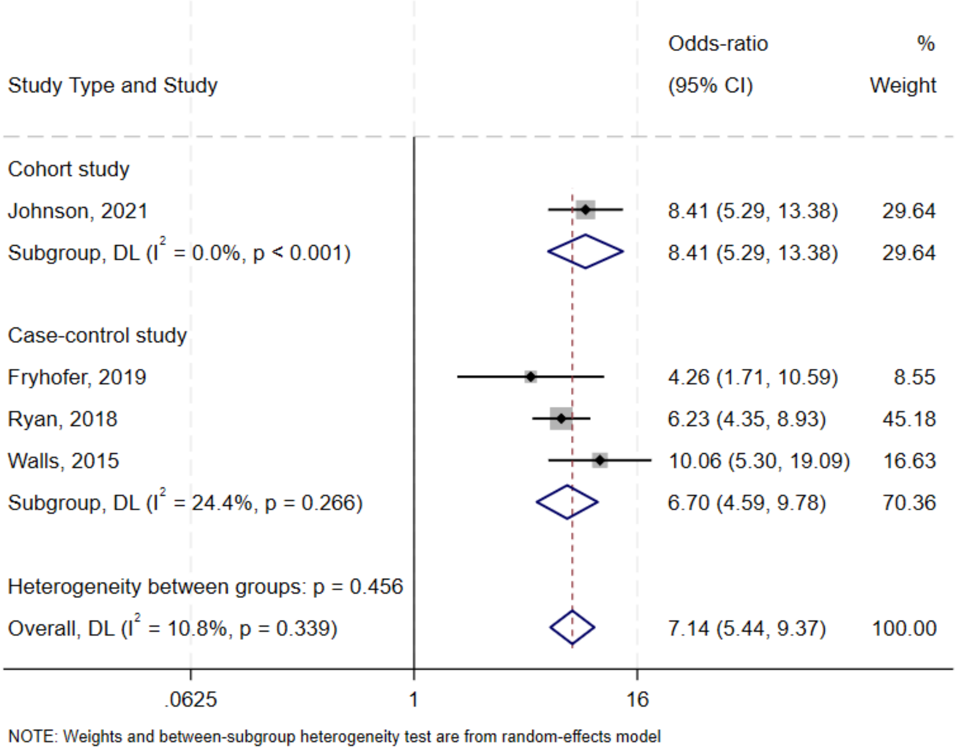

Supplemental Table 1. Summary of the impact of preoperative hypoalbuminemia on clinical outcomes after primary total hip or knee arthroplasty

Reference	Outcomes assessed	Summary of postoperative outcomes between groups
Elliott, 2023 [10]	Unplanned readmission	Patients with preoperative hypoalbuminemia are more likely to experience unplanned readmissions
Lung, 2023 [17]	Periprosthetic dislocation	Patients with preoperative hypoalbuminemia are at a higher risk of experiencing periprosthetic dislocation
Lung, 2023 [18]	Periprosthetic fractures	Patients with preoperative hypoalbuminemia are at a higher risk of experiencing periprosthetic fractures
Abella, 2023 [11]	Unplanned Readmission	Patients with preoperative hypoalbuminemia are at a higher risk of unplanned readmission
Johnson, 2021 [19]	All-cause complications, Unplanned readmission, Mortality	Patients with preoperative hypoalbuminemia have a higher incidence of unplanned readmission, mortality and all-cause complications
Newman, 2020 [5]	All-cause complications, Pneumonia, Sepsis, Septic shock, Urinary tract infection, Superficial incisional infection, Prosthetic joint infections, Stroke, Cardiac arrest, Myocardial infarction, Acute renal failure, DVT, Pulmonary embolism, Wound dehiscence, Transfusion, Reoperation	Patients with preoperative hypoalbuminemia are at a higher risk for all of these adverse outcomes
Guo, 2020 [16]	Prosthetic joint infections	An increased risk of prosthetic joint infections in patients with preoperative hypoalbuminemia.
Sloan, 2020 [21]	Unplanned readmission, Unplanned reoperation	Patients with preoperative hypoalbuminemia are at an increased risk of unplanned readmission and reoperation.
Man, 2020 [9]	Prosthetic joint infections	Patients with preoperative hypoalbuminemia are at a higher risk of experiencing prosthetic joint infections
Fryhofer, 2019 [22]	Mortality, Unplanned reoperation, Unplanned readmission,	Preoperative hypoalbuminemia is associated with an increased risk of morbidity, unplanned readmissions, and the need for reoperations.

Ryan, 2018 [8]	All-cause complications, Pneumonia, Sepsis, Urinary tract infection, Superficial incisional infections, Prosthetic joint infections, Myocardial infarction, DVT/PE, Wound dehiscence, Transfusion, Mortality, Unplanned Reoperation, Unplanned readmission	Preoperative hypoalbuminemia is associated with an increased risk of all of these adverse outcomes.
Haro-Gómez, 2018 [23]	DVT/PE, Unplanned readmission	Preoperative hypoalbuminemia was not significantly associated with DVT/PE and unplanned readmission
Bohl, 2016 [20]	Pneumonia, Superficial incisional infection	Patients with hypoalbuminemia had a higher risk for pneumonia, and superficial incisional infection.
Walls, 2015 [24]	All-cause complications, Pneumonia, Sepsis, Septic shock, Urinary tract infection, Superficial incisional infections, Prosthetic joint infections, Stroke, Myocardial infarction, Acute renal failure, DVT, Pulmonary embolism, Wound dehiscence, Mortality	Patients with hypoalbuminemia had a higher risk for pneumonia, urinary tract infection, superficial incisional infections, prosthetic joint infections, mortality, sepsis, and wound dehiscence, but no differences in septic shock, Stroke, myocardial infarction, acute renal failure, DVT, pulmonary embolism between groups

Supplemental Table 2. Summary of assessment of evidence quality for associations between preoperative hypoalbuminemia and clinical outcomes after total hip or knee arthroplasty *

Outcomes	Illustrative comparative risks* (95% CI)		Relative effect (95% CI)	No of Participants (studies)	Quality of the evidence (GRADE)	Comments
	Assumed risk	Corresponding risk				
	**Normal albumin**	**Preoperative hypoalbuminemia**				
**All-cause complications**	**Study population**		**OR 2.89** (1.94 to 4.31)	196883 (4 studies)	⊕⊖⊖⊖ **very low**[1,2,3,4]	
	**169 per 1000**	**369 per 1000** (282 to 466)				
**Pneumonia**	**Study population**		**OR 3.81** (3.14 to 4.67)	203920 (4 studies)	⊕⊕⊖⊖ **low**[2,3]	
	**3 per 1000**	**22 per 1000** (18 to 26)				
**Sepsis**	**Study population**		**OR 2.54** (1.88 to 3.45)	154317 (3 studies)	⊕⊕⊖⊖ **low**[2,3]	
	**3 per 1000**	**6 per 1000** (5 to 9)				
**Septic shock**	**Study population**		**OR 3.44** (1.15 to 10.25)	25905 (2 studies)	⊕⊕⊖⊖ **low**[2,3,4]	
		**1 per 1000** (0 to 4)				
**Urinary tract infection**	**Study population**		**OR 1.59** (1.13 to 2.23)	154317 (3 studies)	⊕⊖⊖⊖ **very low**[2,5]	
	**10 per 1000**	**17 per 1000** (12 to 23)				
**Superficial incisional SSI**	**Study population**		**OR 2.16** (1.56 to 3.00)	203920 (4 studies)	⊕⊖⊖⊖ **very low**[2,5]	
	**10 per 1000**	**22 per 1000** (16 to 30)				
**PJI**	**Study population**		**OR 4.03** (2.15 to 7.53)	155229 (5 studies)	⊕⊖⊖⊖ **very low**[2,3,5]	
	**2 per 1000**	**9 per 1000** (5 to 17)				
**Stroke**	**Study population**		**OR 2.07** (0.70 to 6.17)	25905 (2 studies)	⊕⊖⊖⊖ **very low**[2,3,6]	
	**1 per 1000**	**2 per 1000** (1 to 5)				
**Cardiac arrest**	See comment	See comment	N/A	0(1 study)	See comment	Data not presented in a format to allow meta-analysis. Studies found that hypoproteinemia was associated with higher risk of cardiac arrest.
**Myocardial infarction**	**Study population**		**OR 2.10** (1.47 to 2.98)	154317 (3 studies)	⊕⊕⊖⊖ **low**[3]	
	**2 per 1000**	**5 per 1000** (3 to 7)				

**Acute renal failure**	**Study population**		**OR 2.41** (0.77 to 7.53)	25905 (2 studies)	⊕⊖⊖⊖ **very low**[2,3,6]	
	**1 per 1000**	**1 per 1000** (0 to 4)				
**Wound dehiscence**	**Study population**		**OR 1.68** (1.06 to 2.69)	154317 (3 studies)	⊕⊖⊖⊖ **very low**[2]	
	**2 per 1000**	**3 per 1000** (2 to 5)				
**Periprosthetic dislocation**	See comment	See comment	N/A	0(1 study)	See comment	Data not presented in a format to allow meta-analysis. Studies found that hypoproteinemia was associated with higher risk of dislocation.
**Periprosthetic fractures**	See comment	See comment	N/A	0(1 study)	See comment	Data not presented in a format to allow meta-analysis. Studies found that hypoproteinemia was associated with higher risk of fractures.
**VTE**	See comment	See comment	Not estimable	0(0)	See comment	Data not presented in a format to allow meta-analysis. Results are inconsistent across studies.
**Unplanned readmission**	**Study population**		**OR 1.14** (0.72 to 1.81)	547760 (7 studies)	⊕⊖⊖⊖ **very low**[1,2,6]	
	**55 per 1000**	**65 per 1000** (42 to 99)				
**Unplanned reoperation**	**Study population**		**OR 1.60** (1.38 to 1.87)	398309 (4 studies)	⊕⊖⊖⊖ **very low**[2]	
	**19 per 1000**	**31 per 1000** (27 to 36)				
**Transfusion**	**Study population**		**OR 1.84** (1.72 to 1.98)	130079 (2 studies)	⊕⊖⊖⊖ **very low**[2]	
	**100 per 1000**	**170 per 1000** (161 to 181)				
**Mortality**	**Study population**		**OR 7.14** (5.44 to 9.37)	371381 (4 studies)	⊕⊕⊕⊖ **moderate**[2,7]	
	**1 per 1000**	**8 per 1000** (6 to 10)				

* The basis for the assumed risk (e.g. the median control group risk across studies) is provided in footnotes. The corresponding risk (and its 95% confidence interval) is based on the assumed risk in the comparison group and the relative effect of the intervention (and its 95% CI). According to the "Grading of recommendation assessment, development, and evaluation" (GRADE) criteria [14].

CI: Confidence interval; **RR:** Risk ratio; **OR:** Odds ratio; **superficial incisional SSI**: superficial incisional surgical site infection; **PJI**: periprosthetic joint infection; **DVT**: deep vein thrombosis; **PE**: pulmonary embolism.

GRADE Working Group grades of evidence

High quality: Further research is very unlikely to change our confidence in the estimate of effect.

Moderate quality: Further research is likely to have an important impact on our confidence in the estimate of effect and may change the estimate.

Low quality: Further research is very likely to have an important impact on our confidence in the estimate of effect and is likely to change the estimate.

Very low quality: We are very uncertain about the estimate.

[1] I^2＞75%

[2] Downgraded by one level for indirectness due to the inclusion of both total hip arthroplasty (THA) and total knee arthroplasty (TKA) populations in the same analysis, which may limit procedure-specific applicability of the findings

[3] OR＞2

[4] No explanation was provided

[5] I^2=50-75%

[6] Downgraded by one level for imprecision due to wide confidence intervals crossing the line of no effect and a limited number of events

[7] OR＞5

Supplemental Table 3. Results of sensitivity analysis (method of "Leave one out")

Studies causing heterogeneity	*OR**	95% *CI**	I^2*
**All to cause complications *OR:* 2.89 (95%*C*I 1.94 to 4.31), I^2=98.1%**			
19	2.46	1.76 to 3.44	95.7%
19, 24	2.08	1.66 to 2.61	74.1%
**Superficial incisional infection *OR:* 2.16 (95%*CI*: 1.56 to 3.00), I^2=60.1%**			
5	2.29	1.74 to 3.02	53.9%
5, 8	2.60	2.03 to 3.32	0%
**Urinary tract infection *OR:* 1.59 (95%*CI*: 1.13 to 2.23), I^2=62.3%**			
24	1.38	0.98 to 1.94	39.6%
**Periprosthetic joint infection *OR:* 4.03 (95%*CI*: 2.15 to 7.53), I^2 = 69.8%**			
16	3.19	1.70 to 5.98	49.8%
9,16	2.65	1.89 to 3.71	0%
**Unplanned reoperation *OR*: 1.60 (95%*CI*: 1.38 to 1.87), I^2 =30.4%**			
22	1.72	1.49 to 1.98	0%
**Unplanned readmission *OR*: 1.14 (95%*CI*: 0.72 to 1.81), I^2 = 98%**			
11	1.94	1.64 to 2.29	84.6%
11, 22	2.06	1.76 to 2.41	78.3%
11, 21, 22	2.20	2.01 to 2.42	29.2%

Supplemental Table 4. Results of sensitivity analysis (Hartung-Knapp correction *versus* Wald-type)

Complication		*OR* (95% *CI*)	
		Wald-type method	Hartung-Knapp method
All-cause complications		2.89 (1.94 to 4.31)	2.89 (1.46 to 5.72)
Systemic complications	Myocardial infarction	2.10 (1.47 to 2.98)	2.10 (1.25 to 3.51)
	Pneumonia	3.83 (3.14 to 4.67)	3.83 (3.45 to 4.24)
	Sepsis	2.54 (1.88 to 3.45)	2.54 (2.23 to 2.90)
	Urinary tract infection	1.59 (1.13 to 2.23)	1.59 (0.69 to 3.67)
	Septic shock	3.44 (1.15 to 10.25)	-*
	Stroke	2.07 (0.70 to 6.17)	-*
	Acute renal failure	2.41 (0.77 to 7.53)	-*
Surgical complications	Periprosthetic joint infection	4.03 (2.15 to 7.53)	4.03 (1.46 to 11.10)
	Superficial incisional infection	2.16 (1.56 to 3.00)	2.16 (1.14 to 4.11)
	Wound dehiscence	1.68 (1.06 to 2.69)	1.68 (0.91 to 3.13)
Additional outcomes	Mortality	7.14 (5.44 to 9.37)	7.14 (4.57 to 11.14)
	Unplanned readmission	1.14 (0.70 to 1.81)	1.14 (0.30 to 4.41)
	Unplanned reoperation	1.60 (1.38 to 1.87)	1.60 (1.26 to 2.04)
	Transfusion	1.84 (1.72 to 1.98)	-*

*: The Hartung-Knapp method is inappropriate in the situations where the sample size amounts to two (specifically, when two studies are incorporated into the meta-analysis).
